# Protective role of the planetary health diet index against metabolic dysfunction-associated steatotic liver disease: global and individual evidence

**DOI:** 10.3389/fnut.2025.1673662

**Published:** 2025-11-14

**Authors:** Zhuo-Qi Liu, Ben-Gang Zhou, Jing-Wen Fang, Yue Yu, Xin Jiang, Xu-Yu Chen, Wen-Jun Wang, Xin-Yu Fu, Jian-Lei Xia, Bang-Jie Liu, Tian-Ming Guo, Min Zhang, Yan-Bing Ding

**Affiliations:** 1 Department of Gastroenterology, The Affiliated Hospital of Yangzhou University, Yangzhou University, Yangzhou, China; 2 Department of Gastroenterology, Northern Jiangsu People’s Hospital, Yangzhou, Jiangsu, China

**Keywords:** planetary health diet index, metabolic dysfunction-associated steatotic liver disease, Global Burden of Disease, Global Dietary Database, National Health and Nutrition Examination Survey

## Abstract

**Background:**

The rising global epidemic of metabolic dysfunction-associated steatotic liver disease (MASLD), coupled with the urgent need for sustainable food systems, highlights the importance of dietary approaches that support both human well-being and environmental resilience. This research examined the correlation between compliance with the Planetary Health Diet Index (PHDI) and MASLD.

**Methods:**

A multi-level analytical framework was adopted to investigate the association between the PHDI and MASLD. At the country level, we applied generalized additive mixed models (GAMMs) to longitudinal data from the Global Dietary Database (GDD) and the Global Burden of Disease (GBD) study (1990–2018) to assess dynamic temporal trends. For individual-level analyses, multivariable regression models were used with data from the National Health and Nutrition Examination Survey (NHANES), adjusting for potential confounding variables.

**Results:**

From 1990 to 2018, PHDI scores exhibited variation across demographic subgroups. Progressive improvements were observed among older adults (≥ 75 years), urban residents, and higher-educated groups. Notably, women consistently demonstrated higher adherence than men. The GAMMs analysis indicated a non-linear association between country-level PHDI and incidence of MASLD, exhibiting a U-shaped partial effect curve. After adjusting for confounders, the protective association reached its maximum at a PHDI of 50.69. In contrast, individual-level analyses revealed a linear inverse relationship between PHDI and MASLD.

**Conclusion:**

This study integrated global and individual-level data to elucidate the association between PHDI and MASLD, revealing reduced adherence among specific sociodemographic groups. These findings underscored the necessity of targeted public health interventions and further longitudinal research to establish causal relationships and develop culturally adapted implementation strategies.

## 1 Introduction

The Planetary Health Diet Index (PHDI) is a quantitative tool designed to operationalize the dietary framework proposed by the EAT-Lancet Commission on Healthy Diets from Sustainable Food Systems (1). This reference diet is specifically formulated to support human health while ensuring that food production remains within planetary ecological limits. The PHDI evaluates adherence across 14 key food groups and nutrients. It encourages high consumption of fruits, vegetables, whole grains, legumes, nuts, and unsaturated oils, while strongly discouraging the intake of red meat, processed foods, added sugar, and starchy vegetables such as potatoes (2, 3). Higher PHDI scores reflect greater alignment with this dual framework of health and sustainability.

Research has indicated that following the PHDI is significantly linked to a lower risk of major chronic diseases in prospective cohort studies. Higher PHDI scores have been consistently linked to lower rates of cardiovascular disease (CVD) (4, 5), type 2 diabetes (T2DM) (6), colorectal cancer (7), and all-cause mortality (8). These protective effects are attributed to the synergistic components of the diet: high fiber, antioxidants, and unsaturated fats that promote cardiometabolic health, combined with limited intake of saturated fats, sodium, and refined carbohydrates that help reduce inflammation and metabolic dysfunction—key mechanisms in the development of chronic diseases (9–12).

Metabolic dysfunction-associated steatotic liver disease (MASLD), which affected approximately 30% of adults worldwide (13), is a significant metabolic disorder whose prevalence mirrors the increasing prevalence of obesity and T2DM. The progression of MASLD can lead to severe complications, including cirrhosis, hepatocellular carcinoma (HCC), and heightened cardiovascular mortality (14, 15). Currently, no pharmacotherapies are approved for the treatment of MASLD, making lifestyle interventions—particularly dietary changes—the primary approach to its management (16, 17). Well-established dietary patterns that protect against MASLD, such as the Mediterranean diet (MEDI) and the Dietary Approaches to Stop Hypertension (DASH), share key principles with the PHDI. These include prioritizing plant-based foods, healthy fats, and minimizing intake of processed sugar, refined grains, and red meat (18).

Despite the PHDI’s structural similarity to MASLD-preventive diets and its demonstrated benefits for related cardiometabolic conditions (CVD, T2DM), its specific relationship with the risk of MASLD has not been sufficiently explored. Therefore, this research seeks to investigate this critical research gap by examining the association between PHDI adherence and MASLD incorporating multi-level evidence—both at the global population level and the individual level. Our objective is to generate robust evidence to inform dietary guidelines that simultaneously promote metabolic liver health and environmental sustainability.

## Materials and methods

### 2.1 Data sources

#### 2.1.1 Global Burden of Disease database

Data on MASLD epidemiology and population statistics were sourced from the Global Health Data Exchange (GHDx). The dataset covered 204 countries and territories from 1990 to 2018, broken down by sex (male/female) and 5-year age groups (15 sex (male/…, ≥ 95 years). GBD 2021 offered a global analysis of 371 diseases, 288 mortality causes, and 88 risk factors (19).

#### 2.1.2 Global Dietary Database

To assess potential dietary determinants of MASLD patterns, we analyzed data from the GDD 2018 (20). The GDD compiled nationally representative dietary surveys from 185 countries (1990–2018) using a Bayesian hierarchical modeling approach. Standardized dietary tools, including 24-h recalls and food frequency questionnaires, were used to collect data (21). Intakes were categorized by age, sex, education, and residence (22). To ensure demographic consistency with the GBD data, our analysis was limited to individuals aged 15 years and older. Age-specific energy intake guidelines established limits of 2000 kcal/day for individuals aged 15–74 and 1700 kcal/day for those aged 75 and older (23).

#### 2.1.3 National Health and Nutrition Examination Survey

Individual-level data were sourced from the NHANES, administered by the National Center for Health Statistics (NCHS) under the Centers for Disease Control and Prevention (CDC). As a cross-sectional study, NHANES evaluates the health and nutritional status of the non-institutionalized civilian population in the United States through in-person interviews and medical examinations carried out in mobile examination centers. We analyzed data from 10 NHANES cycles (1999–2000 through 2017–2018), culminating in a final analytical cohort of 7,758 participants. Following NHANES analytical guidelines, the study incorporated sample weights, pseudo-primary sampling units (sdmvpsu), and pseudostrata (sdmvstra) to adjust for the complex, stratified, multistage sampling methodology. Sample weights were computed as 1/5 × WTSAF4YR for the combined 1999–2002 cycles and 1/10 × WTSAF2YR for the 2003–2018 cycles.

### 2.2 Dietary assessment

The PHDI assessed intake levels of 14 food groups within the GDD: whole fruits, whole grains, nuts and seeds, non-starchy vegetables, legumes and soy food, unsaturated oils, fish, starchy vegetables, dairy, eggs, red and processed meat, poultry, saturated oils, and added sugar (2, 3). Each component received a score ranging from 0 to 10 points, indicating the extent to which the recommended intake guidelines were followed. Dietary data were adjusted to a standard energy intake of 2,500 kcal/day through the residual method. Due to the absence of poultry intake data in the GDD, the maximum attainable PHDI score for the global analysis was capped at 130 points (Supplementary Table 1). For the US-specific analysis using NHANES data, a modified version of the index—PHDI-US—was developed, incorporating two additional metrics related to vegetable diversity: the proportion of dark green and red/orange vegetables. This adaptation expanded the index to 16 components, with each of the two proportion-based metrics scored from 0 to 5, resulting in a maximum total score of 150 points (Supplementary Table 2). The adapted PHDI-US has been validated in US populations and demonstrates closer alignment with the EAT-Lancet dietary guidelines (24, 25).

### 2.3 Metabolic dysfunction-associated steatotic liver disease defintion

Global MASLD incidence data were drawn from GBD 2021 estimates generated by DisMod-MR 2.1, a Bayesian meta-regression method applied consistently across all regions and time periods (26, 27). Within the NHANES dataset, MASLD status was determined using the US Fatty Liver Index (US-FLI), calculated as follows: US-FLI = (e*^y^*)/(1 + e*^y^*) × 100, where y = -0.8073 × Non-Hispanic Black + 0.3458 × Mexican American + 0.0093 × age + 0.6151 × ln(GGT) + 0.0249 × waist circumference + 1.1792 × ln(insulin) + 0.8242 × ln(glucose) − 14.7812. Ethnicity variables were dichotomized (1 = yes, 0 = no). Individuals with a US-FLI score of 30 or higher were categorized as having MASLD, if they had negative viral hepatitis test results and did not consume excessive amounts of alcohol, defined as more than 2 drinks per day for men and 1 drink per day for women (28, 29).

### 2.4 Covariates

Within the global analysis investigating the link between national PHDI levels and the incidence of MASLD, adjustments were restricted to age, sex, year, and population due to data limitations. For the individual-level analysis within NHANES, covariates included age, sex, race, marital status, education, family income, smoking, alcohol use, BMI, physical activity, hypertension, and diabetes. Race was classified into the following categories: Mexican American, Other Hispanic, Non-Hispanic White, Non-Hispanic Black, and Other Race. Educational attainment was divided into three levels: less than high school, high school graduate or equivalent, and beyond high school (30). Family income was classified using the Poverty Income Ratio (PIR) into three levels: low (≤ 1.3), medium (1.3–3.5), and high (> 3.5). Smoking and alcohol use were based on behavioral history, while hypertension and diabetes were determined by self-reported physician diagnosis. Physical activity was assessed through the calculation of the weekly duration of occupational, household, leisure, and commuting activities, with results reported in MET-min/week (31).

### 2.5 Statistical analysis

We examined global temporal trends in the PHDI from 1990 to 2018 across various demographic strata, investigating their associations with MASLD incidence using both cross-sectional and longitudinal analytical approaches. In the 2018 cross-sectional analysis, population-weighted bubble plots with LOESS smoothing (95% CIs) were employed to visualize country-level relationships (32, 33). For longitudinal analyses, we constructed three hierarchical models using generalized additive mixed modeling (GAMM): (1) a linear mixed model including fixed effects for PHDI, sex, age, population, and year, along with random location intercepts; (2) a semiparametric model that combined linear PHDI effects with non-linear cubic regression splines for age, population, and year; and (3) a fully non-linear model extending spline terms to all continuous predictors. The GAMM framework was chosen to model complex non-linear relationships and account for the hierarchical structure of our longitudinal global data. It captures temporal trends flexibly without restrictive parametric assumptions and handles spatial clustering through location-level random effects (34). All models incorporated analytic weights based on population size and were fitted using restricted maximum likelihood (REML) estimation with computational acceleration techniques. Spatial clustering was accounted for through random intercepts at the location level, and year variables were median-centered to enhance interpretability. Model selection was based on comparisons of Bayesian Information Criterion (BIC), Akaike Information Criterion (AIC), and the percentage of deviance explained. Non-linear effects were formally tested using spline-specific *p*-values.

The NHANES data were described using weighted means ± standard deviation (SD) for continuous variables, while categorical variables were presented using unweighted counts and weighted percentages. To investigate the connection between PHDI and MASLD, survey-weighted logistic regression was employed, with results expressed as odds ratios (ORs) and 95% confidence intervals (CIs). Additionally, a restricted cubic spline (RCS) regression model incorporating four knots located at the 5th, 35th, 65th, and 95th percentiles was applied within fully adjusted models to assess potential non-linear associations. All statistical analyses and graphical representations were conducted using R software (version 4.2.2).

## 3 Results

### 3.1 Demographic trends in PHDI

Between 1990 and 2018, the PHDI demonstrated divergent temporal trends across various demographic subgroups (Supplementary Table 3). Specifically, individuals aged 75 years and older exhibited a consistent upward trajectory in PHDI scores over time, whereas younger age groups did not display statistically significant changes. Urban populations experienced a significant annual increase of 0.206 (*P* = 0.007), in contrast to rural populations, which showed a significant annual decline of −0.239 (*P* = 0.008). Educational attainment was a key determinant of these trends; individuals with more than 12 years of education exhibited an annual increase of 0.45 (*P* < 0.001), while no notable changes were observed among individuals with lower levels of education. Furthermore, females consistently achieved higher PHDI scores than males across all demographic categories, and this gender difference remained stable across age groups, education levels, and residential settings.

### 3.2 Geographical and dietary composition disparities in PHDI

**FIGURE 1:**
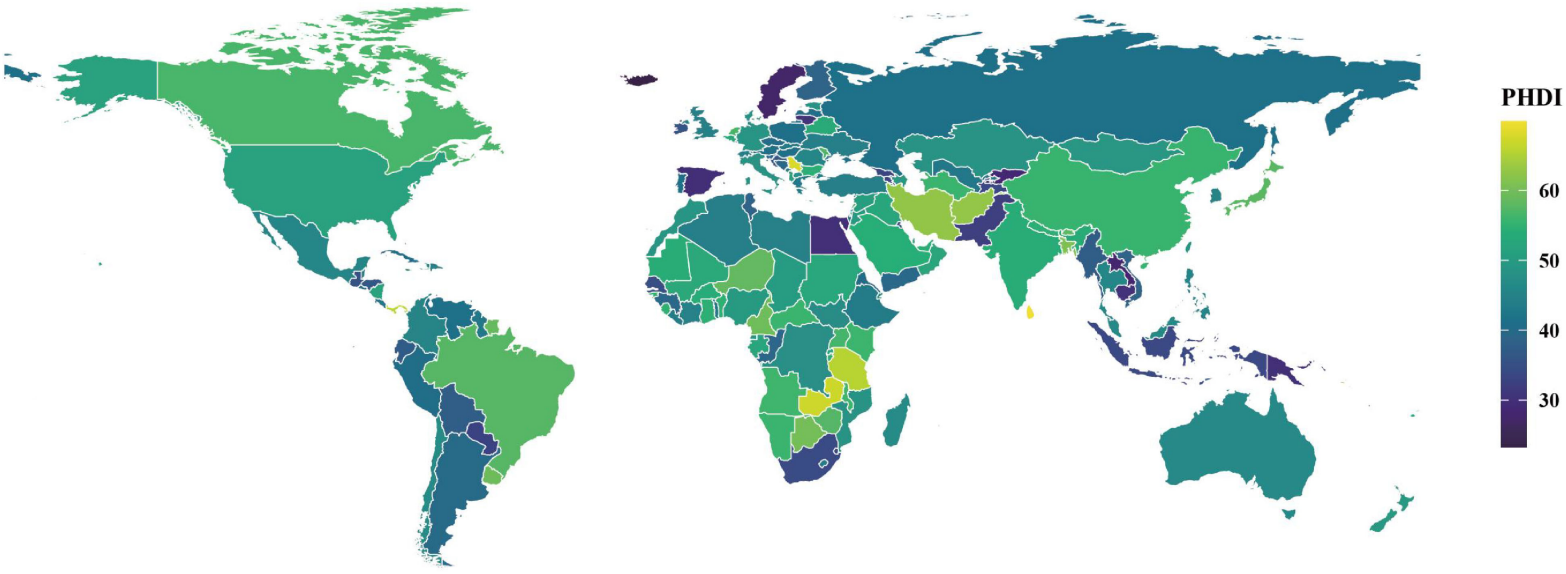
Distribution of PHDI scores among populations aged 15 years or older in 185 countries, 2018.

In 2018, PHDI scores demonstrated considerable global variation (Figure 1; Supplementary Table 4). The Democratic Socialist Republic of Sri Lanka achieved the highest score (69.01), followed by the Independent State of Samoa (68.25) and the Republic of Serbia (64.26). In contrast, the Republic of Iceland recorded the lowest PHDI score (26.95), with similarly low values observed in the Kingdom of Sweden (30.45) and the Lao People’s Democratic Republic (31.52). An analysis of individual dietary component scores revealed that non-starchy vegetables and unsaturated oils were the highest-performing food groups, whereas red and processed meat, eggs, saturated oils, and added sugar received significantly lower scores (Supplementary Table 5).

### 3.3 Association between PHDI and incidence of MASLD

**FIGURE 2:**
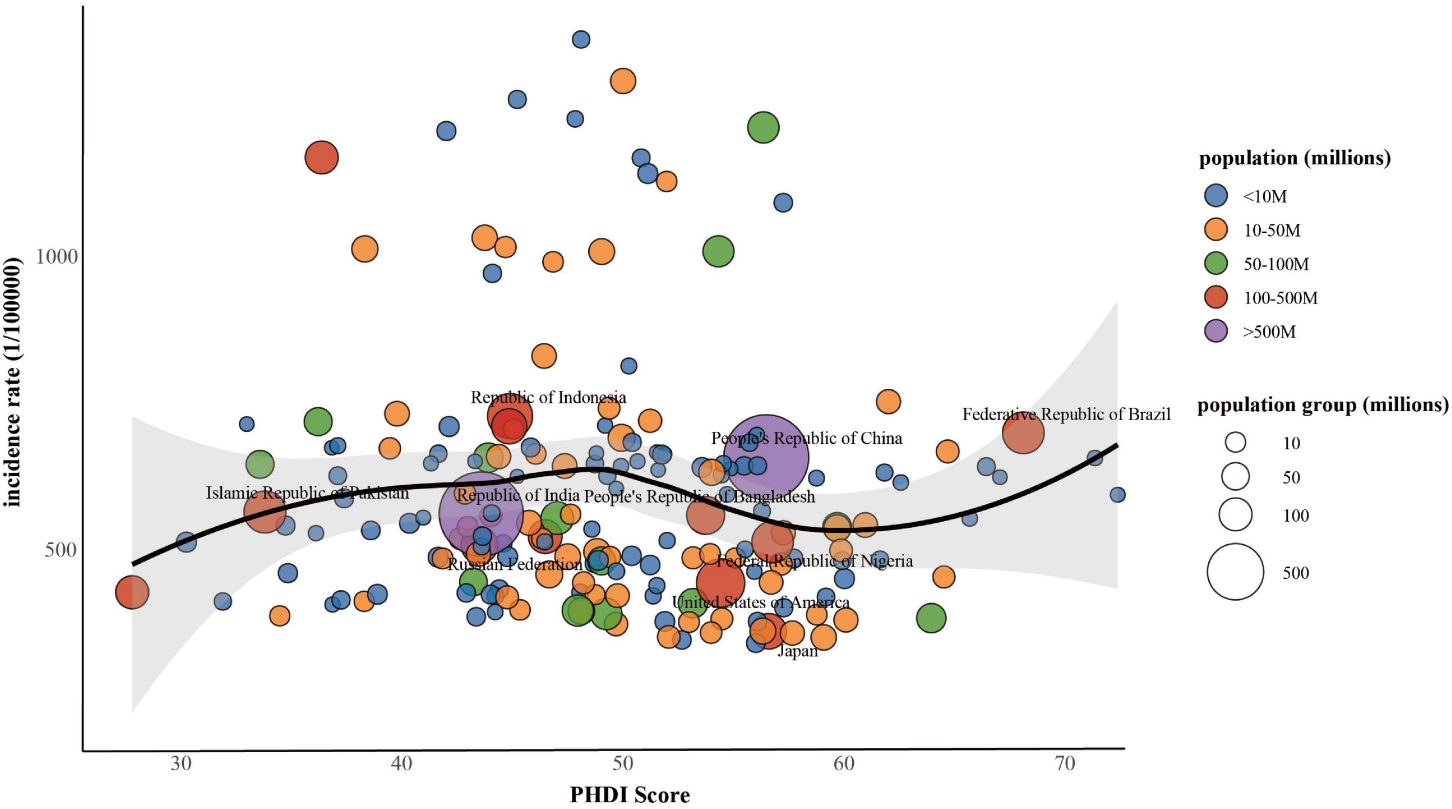
Associations between PHDI and incidence of MASLD among populations aged 15 years or older, 2018.

**FIGURE 3:**
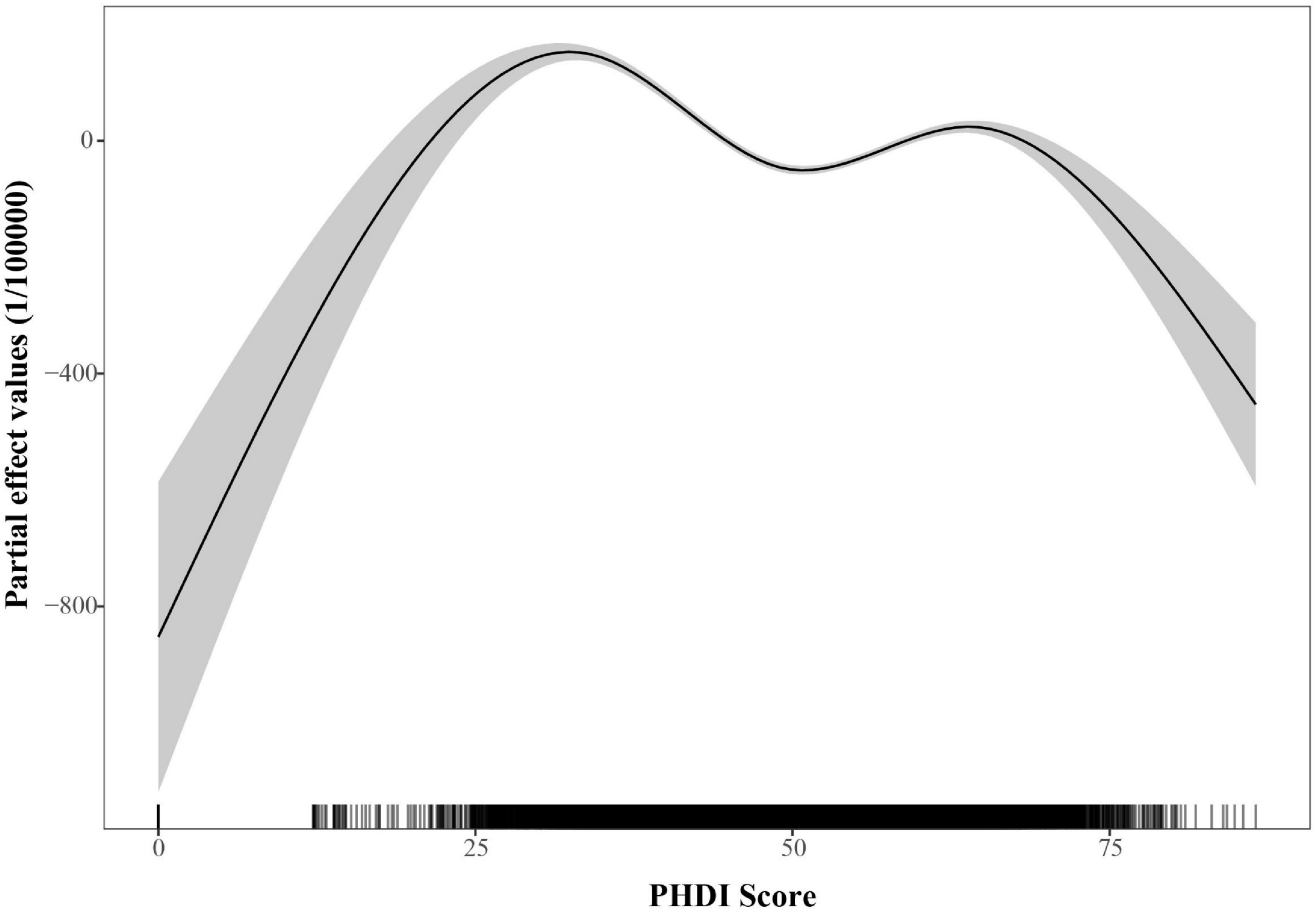
Partial effects of PHDI on the incidence of MASLD within the generalized additive mixed model (GAMM).

Figure 2 presented the connection between the PHDI and the incidence of MASLD in 2018. Initial LOESS regression analysis indicated no significant non-linear trends. Among the three competing models, the fully non-linear GAM exhibited the best performance, with a BIC of 733,584.43 and explaining 33.1% of the deviance. This model outperformed both the semiparametric GAM (BIC = 734,407.89) and the linear mixed model (BIC = 739,833.44), as summarized in Table 1. The analysis identified a significant sexual dimorphism, with females demonstrating a substantially lower MASLD incidence (β = –91.581, *P* < 0.001). All smooth terms displayed statistically significant non-linear associations (*P* < 0.001), as detailed in Table 2, particularly revealing a U-shaped relationship between PHDI and MASLD incidence. Within most of the PHDI range (scores 44.77–58.95), incidence rates remained below the population-average level, with the strongest protective effect observed at a PHDI score of 50.69 (partial effect = –50.75), as illustrated in Figure 3.

**TABLE 1:** Statistical modeling of PHDI and incidence of MASLD.

Model Type	AIC	BIC	Deviance explained (%)
Linear	739772.59	739833.44	22.67
Semiparametric	734268.85	734407.89	31.78
Full non-linear	733419.29	733584.43	33.10

AIC, Akaike Information Criterion; BIC, Bayesian Information Criterion.

**TABLE 2:** Mixed-effect modeling results for PHDI and incidence of MASLD.

Variables	Type	Estimate (β)	SE	EDF	*P*
(Intercept)	Parametric	636.127	8.968	–	< 0.001
Sex (female)	Parametric	−91.581	2.649	–	< 0.001
s(PHDI)	Smooth	–	–	3.978	< 0.001
s(age)	Smooth	–	–	3.992	< 0.001
s(population)	Smooth	–	–	4.970	< 0.001
s(year)	Smooth	–	–	1.971	< 0.001
Country	Random	–	–	0.993	< 0.001

Fixed effects are presented as parametric coefficients (β) with standard errors (SE). Smooth terms [denoted as s(variable)] represent non-linear relationships, with effective degrees of freedom (EDF) indicating function complexity (EDF = 1 indicates linearity).

### 3.4 NHANES analysis

**FIGURE 4:**
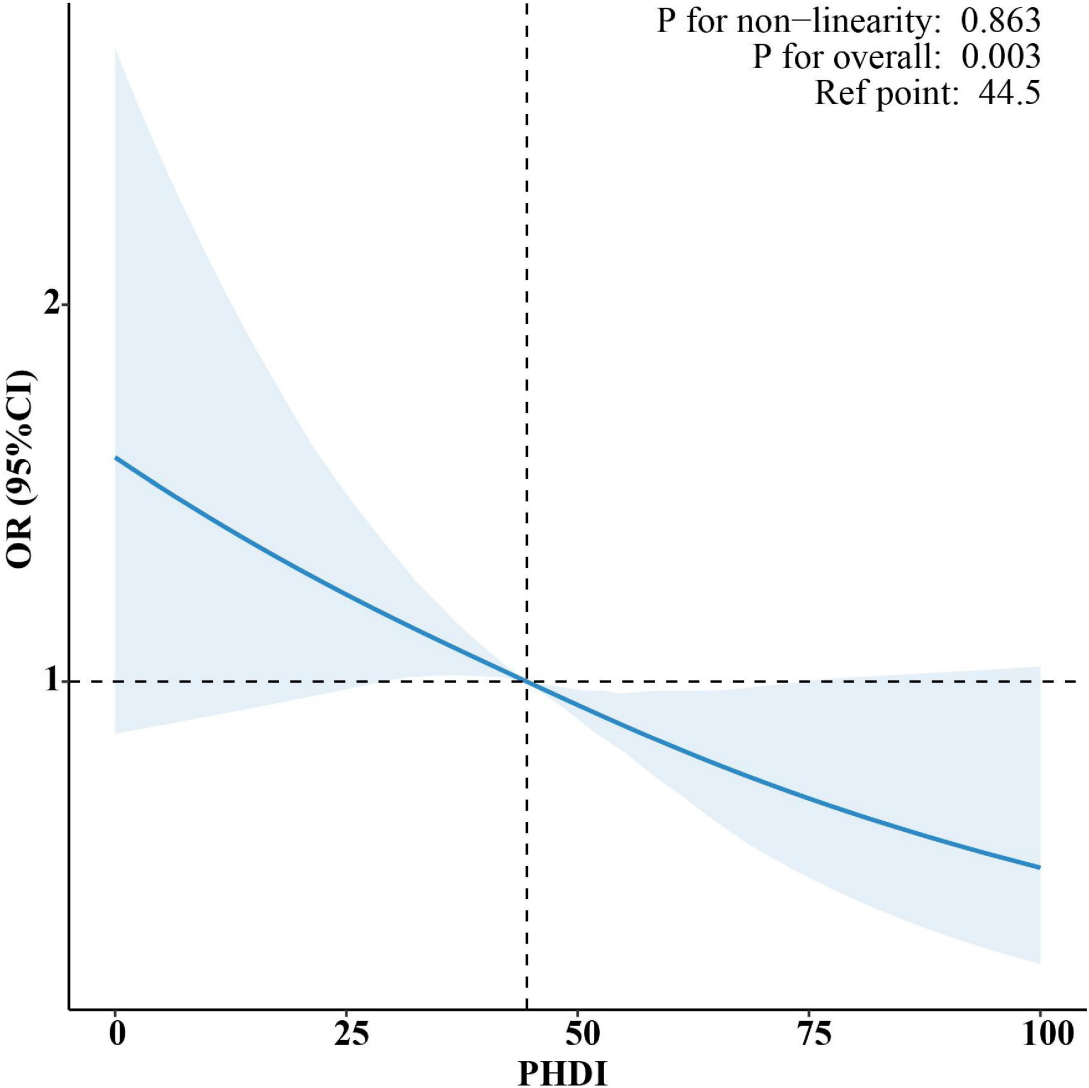
Restricted cubic spline (RCS) analysis of the non-linear association between PHDI and risk of MASLD.

Participant selection followed the flow chart detailed in Supplementary Figure 1. The NHANES cohort had a mean weighted age of 49.67 years, with 54.22% of participants identifying as female (Supplementary Table 6). Higher PHDI scores were significantly associated with lower risk of MASLD in Table 3. Specifically, for every one-point increase in PHDI score, there was a 1.1% decrease in risk for MASLD in the fully adjusted model (95% CI: 0.982–0.995; *P* < 0.001). When stratified by quartiles, individuals in the top quartile (Q4) demonstrated a 37.3% reduced likelihood of developing MASLD in comparison to those in the bottom quartile (Q1) (95% CI: 0.488–0.805; *P* < 0.001). Furthermore, RCS analysis did not detect any statistically significant non-linear associations (*P* for non-linearity > 0.05) (Figure 4).

**TABLE 3:** Associations between PHDI and MASLD.

Variables	Model 1*^a^*		Model 2*^b^*		Model 3*^c^*	
	OR (95% CI)	*P*	OR (95% CI)	*P*	OR (95% CI)	*P*
PHDI	0.983(0.978, 0.988)	< 0.001	0.978(0.973, 0.984)	< 0.001	0.989(0.982, 0.995)	< 0.001
PHDI						
T1	Ref		Ref		Ref	
T2	0.863(0.732, 1.017)	0.078	0.743(0.619, 0.893)	0.002	0.690(0.532, 0.895)	0.006
T3	0.816(0.693, 0.961)	0.015	0.710(0.599, 0.842)	< 0.001	0.805(0.643, 1.009)	0.060
T4	0.541(0.455, 0.644)	< 0.001	0.452(0.373, 0.547)	< 0.001	0.627(0.488, 0.805)	< 0.001
Trend test		< 0.001		<0.001		0.002

^a^Model 1: Adjusted for no covariates.

^b^Model 2: Adjusted for age, gender, race, marital status, PIR and education.

^c^Model 3: Further adjusted BMI, MET, smoking, alcohol use, hypertension, diabetes.

## 4 Discussion

This study presented a comprehensive evaluation of PHDI and its association with MASLD using a multi-level analytical framework. The findings provided robust evidence in support of dietary interventions that simultaneously promote human health and planetary sustainability, while advancing the mechanistic understanding of sustainable dietary patterns.

Demographic analyses revealed significantly higher adherence to the PHDI among women, older adults, urban populations, and individuals with higher educational attainment. These differences could be attributed to a range of sociobehavioral and economic factors. Gender differences were associated with women’s greater health awareness and primary responsibility for food preparation, resulting in higher vegetable consumption (35, 36). Although men showed slightly higher fish consumption, their significantly greater intake of red meat may counteract potential health benefits through pro-inflammatory effects, while also increasing cardiometabolic risks (37, 38). Higher PHDI scores in older adults were largely driven by economic benefits greater intake -based food consumption can save up to 21% in costs. Plant-predominant diets also provided anti-inflammatory effects through polyphenols and dietary fiber, offering dual advantages for both affordability and health (39, 40). Urban-rural disparities reflected structural inequities: urban populations had superior access to diverse and high-quality foods, whereas rural diets frequently lacked essential PHDI components such as nuts and legumes (41). The role of education extended beyond nutritional knowledge; it influenced intentional and sustainable food choices. Individuals with higher education demonstrated a stronger commitment to reducing meat consumption (42–44). These findings underscored the necessity of demographically tailored interventions. Key strategies should include enhancing nutrition education programs specifically designed for men, improving both physical and economic access to diverse plant-based foods in rural areas, and implementing subsidy initiatives to increase the affordability of healthy plant-based options for elderly and low-income populations.

This study extended prior evidence (30) on the association between PHDI and MASLD through a multilevel analytical framework. The U.S. analysis confirmed a robust linear inverse association through the application of adapted methodologies (PHDI-US and US-FLI), thereby reinforcing the clinical relevance of earlier NHANES-based evidence. These findings align with international evidence: Iran’s RaNCD cohort reported an association between greater compliance with plant-based dietary patterns and a lower likelihood of hepatic fibrosis (45). Similarly, the UK Biobank study found that optimal plant-based dietary patterns were associated with a lower incidence of MASLD and reduced hepatic fat content, whereas unhealthy plant-based patterns were linked to higher risks (46). Notably, longitudinal analysis of global data demonstrated a non-linear association between PHDI and incidence of MASLD, characterized by a U-shaped partial effect curve, with the protective effect peaking at a PHDI value of 50.69. This pattern was ecological in nature and did not reflect individual-level risk. In contrast, NHANES analyses revealed a linear inverse association among U.S. adults, providing more direct individual-level evidence. This divergence arose from key methodological and contextual differences. Methodologically, the analyses differed in their dietary assessment tools, index composition, and capacity for confounder adjustment. These methodological variations, combined with contextual differences between globally heterogeneous dietary patterns and the relatively homogeneous U.S. dietary environment, collectively accounted for the distinct association patterns observed across analytical levels. The U-shaped relationship could be further elucidated by examining dietary quality across the PHDI spectrum. At lower PHDI levels, as was seen in nations with traditional animal-based diets or those reliant on refined carbohydrates, diets were deficient in protective plant compounds, leaving the liver more exposed to pro-inflammatory and lipogenic insults (46–48). Conversely, very high PHDI scores in certain low-income countries reflected nutritionally inadequate “passive” plant-based diets that were driven by economic constraints rather than intentional, health-promoting food choices (49, 50). These findings collectively underscored that the association between PHDI and MASLD was modulated by dietary quality and contextual factors. The observed optimal PHDI score suggested that maximal protection against MASLD was achieved not through extreme adherence to plant-based diets, but at a balanced level of dietary intake. This finding supported the prioritization of high-quality, diverse plant-based dietary patterns as measurable targets for public health policy. Consequently, the study indicated that public health strategies should aim not only to promote plant-based eating, but also to enhance overall diet quality and diversity. The analysis highlighted that implementation must be context-specific: in high-income countries, efforts should focus on shifting consumption patterns, whereas in low-income settings, policies needed to address nutrient inadequacies associated with monotonous, economically constrained plant-based diets. Ultimately, this work demonstrated that the alignment of human and planetary health depended on balanced dietary optimization rather than the pursuit of dietary extremes.

At a mechanistic level, the biological pathways potentially underlying the associations observed at both country and individual levels involve synergistic interactions among three key classes of bioactive plant compounds. Polyphenols found in fruits and vegetables reduce oxidative stress through free radical scavenging, inhibit NF-κB-mediated inflammatory signaling, and activate Nrf2-dependent antioxidant defenses (51). Dietary fibers from whole grains and legumes are fermented by gut microbiota into butyrate, which strengthens the intestinal barrier to attenuate LPS-TLR4-driven inflammation, suppresses excessive immune responses via regulatory T-cell activation, inhibits pro-inflammatory macrophages, enhances insulin signaling through GLP-1 secretion, and exerts direct hepatoprotective anti-inflammatory effects. Meanwhile, unsaturated fats abundant in nuts and seeds activate PPARα to promote mitochondrial β-oxidation and inhibit SREBP-1c-dependent lipogenesis (52–54). This synergistic network operates through three central mechanisms—enhanced antioxidant defense, improved gut-liver axis function, and metabolic reprograming—that collectively reduce hepatic lipid accumulation, alleviate insulin resistance, and interrupt the inflammatory-fibrotic cascade driving MASLD progression (55).

Traditional dietary patterns, such as the Mediterranean, DASH, and HEI-2015 diets, share common goals with the PHDI—promoting consumption of fruits, vegetables, and whole grains while limiting added sugars and saturated fats (56). However, in contrast to the Mediterranean diet, which permits moderate consumption of fish and dairy, or the DASH diet, which includes lean meats, the PHDI introduces a paradigm shift by integrating environmental sustainability principles. This innovative approach sets stringent thresholds: red meat intake is limited to 14 g/day (equivalent to one thin slice of beef), starchy vegetables and added sugars are tightly regulated, and both planetary ecological boundaries and human health outcomes are addressed simultaneously. However, the implementation of this unified framework encounters the challenge of reconciling global standards with local settings, including misalignment with traditional dietary patterns and economic barriers in low-income regions (57, 58). This highlights a central paradox: reconciling global standards with local realities. Future strategies should therefore develop localized adaptations, not rigidly apply universal metrics.

This study had several limitations. First, the observational design, which included both aggregated national-level data from the GBD/GDD and individual-level data from NHANES, did not permit definitive causal inference. Therefore, future longitudinal studies were needed to confirm the causal association between PHDI adherence and MASLD risk. Second, the NHANES-based analysis demonstrated a strong linear inverse association in the U.S. population, but these findings may not be generalizable to populations with different dietary patterns and socioeconomic contexts. Future validation in diverse international cohorts was recommended. Third, in the global analysis, data quality varied across countries, and statistical adjustments were restricted to age, sex, population, and year due to limited availability of covariate data. As a result, key confounders, such as obesity prevalence, physical activity levels, socioeconomic status, and the prevalence of underlying conditions including hypertension and diabetes, were not adjusted for, potentially affecting the observed associations. Fourth, the GDD database exhibited notable coverage gaps, particularly the absence of poultry consumption data and insufficient differentiation between non-soy legumes and soy-based food items, which might have introduced measurement bias into PHDI calculations. Finally, at the individual level, the analysis relied on self-reported dietary intake, which was prone to recall bias, and used the US-FLI as a proxy for MASLD diagnosis, a method that might have entailed misclassification bias relative to imaging or histology-based diagnostic criteria.

## 5 Conclusion

This study employed a multi-level analytical framework to evaluate the association between the PHDI and MASLD, demonstrating that greater adherence was significantly associated with a lower disease risk. Notably, the identified protective threshold of PHDI, together with the linear negative association observed in PHDI-US, collectively indicated that the relationship between PHDI and MASLD was complex and potentially modulated by socioeconomic and cultural dietary contexts. These findings provided empirical support for integrating liver health objectives into sustainable dietary guidelines. Future longitudinal studies are warranted to confirm the causal nature of this association and to elucidate the hepatoprotective effects of the planetary health diet.

## Data Availability

The original contributions presented in the study are included in the article/Supplementary material, further inquiries can be directed to the corresponding authors.
