# Supplementary material for: Protective role of the planetary health diet index against metabolic dysfunction-associated steatotic liver disease: global and individual evidence
Source: Front Nutr. 2025 Nov 14;12:1673662. doi: 10.3389/fnut.2025.1673662 (PMC12661568; doi:10.3389/fnut.2025.1673662)
Supplement: Supplementary file 1 [file Supplementary_file_1.docx]

Supplementary Material

# Supplementary Figures and Tables

## Supplementary Figures


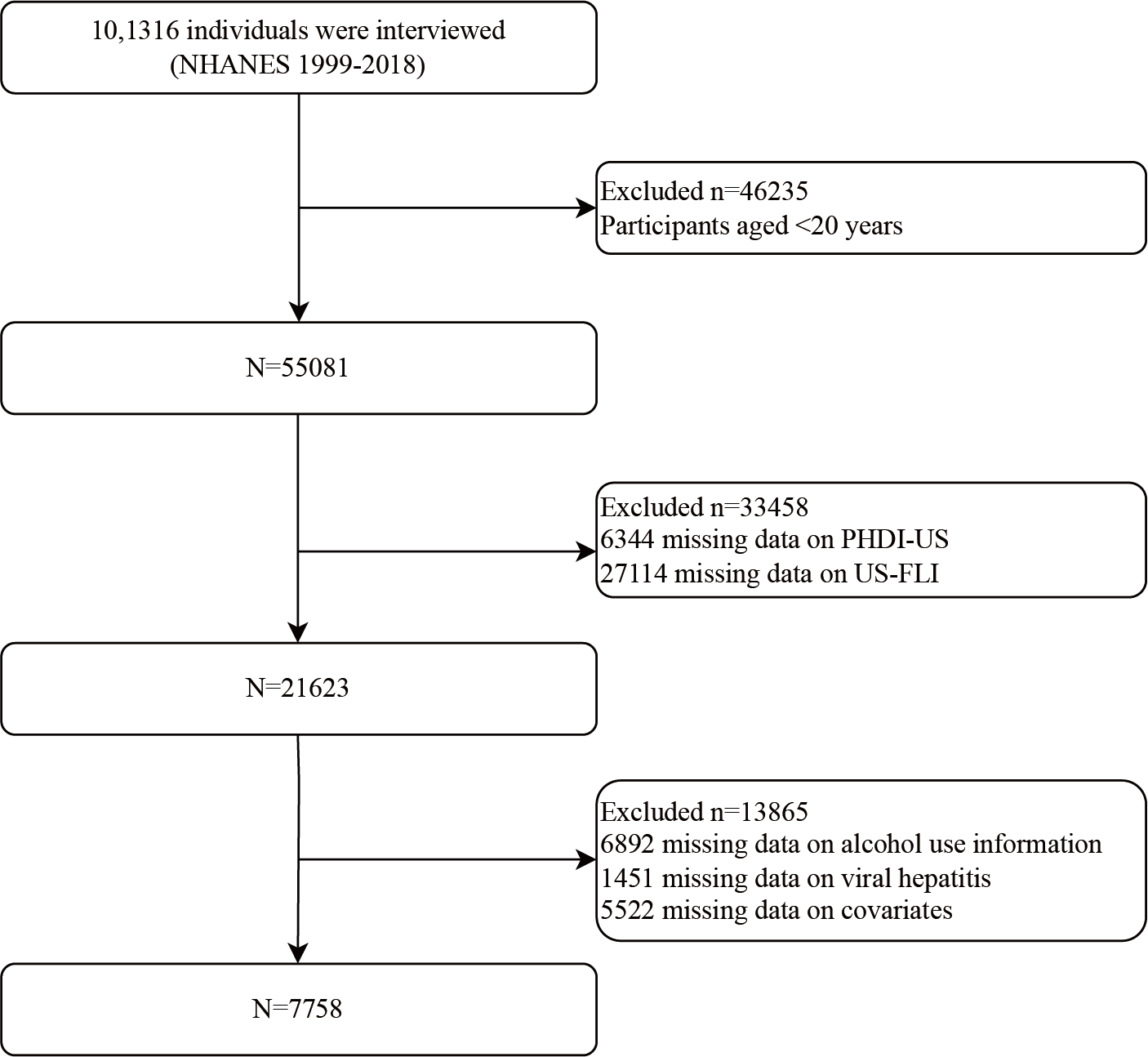


**Supplementary Figure 1** Study flow diagram in the NHANES study

## Supplementary Tables

**Supplementary Table 1** Scoring criteria for the PHDI components in the GDD, standardized to a 2500 kcal/day reference diet

| **Type** | **Dietary component** | **EAT-Lancet recommendations** | **0 points** | **0-10 points** | **10 points** | 1. **points** | **0 points** |
| --- | --- | --- | --- | --- | --- | --- | --- |
| Adequacy components | 1.Whole fruits | 200 (100-300g) | 0g | 0-200g | ≥200g |  |  |
| Adequacy components | 2.Nonstarchy vegetables | 300 (200-600g) | 0g | 0-300g | ≥300g |  |  |
| Adequacy components | 3.Nuts and seeds | 50 (0-75g) | 0g | 0-50g | ≥50g |  |  |
| Adequacy components | 4.Legumes and soy food | 75 (0-150g) | 0g | 0-75g | ≥75g |  |  |
| Adequacy components | 5.Whole grains | 232 (0-464g) | 0g | 0-232g | 232g | 232-464g | ≥464 g |
| Adequacy components | 6.Unsaturated oils | 40 (20-80g) | 0g (or 0% of total energy intake) | 0-40g (0-14% of total energy intake) | 40g (or 14 % of total energy intake) | 40-80g (or 14-28% of total energy intake) | 80g (or 28% of total energy intake) |
| Adequacy components | 7.Fish | 28 (0-100g) | 0g | 0-28 | 28g | 28-100g | ≥100g |
| Moderation components | 8.Starchy vegetables | 50 (0-100g) | 0g | 0-50g | 50 | 50-100g | ≥100g |
| Moderation components | 9.Dairy | 250 (0-500g) | 0g | 0-250g | 250g | 250-500g | ≥500g |
| Moderation components | 10.Poultry | 29 (0-58g) | 0g | 0-29g | 29g | 29-58g | ≥58g |
| Moderation components | 11.Eggs | 13 (0-25g) | 0g | 0-13g | 13g | 13-25g | ≥25g |
| Moderation components | 12.Saturated oils | 11.8 (0-11.8g) |  |  | 0g (or 0% of total energy intake) | 0-11.8g (or 0-3.8% of total energy intake) | ≥11.8g (or ≥3.8% of total energy intake) |
| Moderation components | 13.Added sugar | 31 (0-31g) |  |  | 0g (or 0% of total energy intake) | 0-31g (or 0-5% of total energy intake) | ≥31g (or ≥5% of total energy intake) |
| Moderation components | 14.Red and processed meat | 14 (0-28g) | 0g | 0-14g | 14g | 14-28g | ≥28g |
| The EAT-Lancet reference planetary-health diet recommendations are designed based on a total energy intake of 2500 kcal/day. | | | | | | | |

**Supplementary Table 2** Scoring criteria and component specifications for the PHDI in the NHANES

| **Type** | **Dietary component** | **EAT-Lancet recommendations** | **0 points** | **0-10 points** | **10 points** | **10-0 points** | **0 points** |
| --- | --- | --- | --- | --- | --- | --- | --- |
| Adequacy components | 1.Whole fruits | 200 (100-300g) | 0% | 0-10.1% | ≥10.1% |  |  |
| Adequacy components | 2.Nonstarchy vegetables | 300 (200-600g) | 0% | 0-15.2% | ≥15.2% |  |  |
| Adequacy components | 3.Nuts and seeds | 50 (0-75g) | 0% | 0-2.5% | ≥2.5% |  |  |
| Adequacy components | 4.Legumes and soy food | 75 (0-150g) | 0% | 0-8.5% | ≥8.5% |  |  |
| Adequacy components | 5.Whole grains | 232 (0-464g) | 0% | 0-25.0% | ≥25.0% |  |  |
| Optimum component | 6.Unsaturated oils | 40 (20-80g) | 0% | 0-2.0% | 2.0% | 2.0-4.0% | ≥4.0% |
| Optimum component | 7.Fish | 28 (0-100g) | 0% | 0-1.4% | 1.4% | 1.4-5.1% | ≥5.1% |
| Optimum component | 8.Starchy vegetables | 50 (0-100g) | 0% | 0-2.5% | 2.5% | 2.5-5.1% | ≥5.1% |
| Optimum component | 9.Dairy | 250 (0-500g) | 0% | 0-12.6% | 12.6% | 12.6-25.3% | ≥25.3% |
| Optimum component | 10.Eggs | 13 (0-25g) |  |  | ≤0.7% | 0.7-1.3% | ≥1.3% |
| Moderation components | 11.Poultry | 29 (0-58g) |  |  | ≤1.5% | 1.5-2.9% | ≥2.9% |
| Moderation components | 12.Saturated oils | 11.8 (0-11.8g) |  |  | ≤0.3% | 0.3-0.6% | ≥0.6% |
| Moderation components | 13.Added sugar | 31 (0-31g) |  |  | ≤0.8% | 0.8-1.6% | ≥1.6% |
| Moderation components | 14.Red and processed meat | 14 (0-28g) |  |  | ≤0.7% | 0.7-1.4% | ≥1.4% |
|  |  |  | **0 points** | **0-5 points** | **5 points** | **5-0 points** | **0 points** |
| Ratio component | 15.DGV/total ratio |  | 0% | 0-33.3% | 33.3% | 33.3-100% | 100% |
| Ratio component | 16.Rev/total ratio |  | 0% | 0-33.3% | 33.3% | 33.3-100% | 100% |
| Component intake ranges are represented as the percentage of total grams of foods and beverages (excluding water) consumed.  DGV/total ratio component is the ratio between the grams of dark green vegetables (DGV) consumed and the grams consumed within the Non-starchy vegetables component.  ReV/total ratio component is the ratio between the grams of red and orange vegetables (ReV) consumed and the grams consumed within the Non-starchy vegetables component. | | | | | | | |

**Supplementary Table 3** Temporal trends in PHDI scores stratified by age group, education, and residence, 1990-2018

|  | **1990** | **1995** | **2000** | **2005** | **2010** | **2015** | **2018** | **EAPC** | ***P*** |
| --- | --- | --- | --- | --- | --- | --- | --- | --- | --- |
| **Total** |  |  |  |  |  |  |  |  |  |
| 15-19 years | 49.89 (49.61, 50.73) | 49.35 (49.04, 48.91) | 48.71 (48.77, 48.78) | 47.21 (47.79, 49.40) | 48.83 (47.79, 51.22) | 47.69 (46.93, 50.26) | 48.08 (46.72, 50.70) | -0.134 | 0.068 |
| 20-24 years | 48.79 (50.01, 49.26) | 46.87 (48.94, 45.62) | 47.38 (48.33, 47.68) | 47.32 (48.23, 48.82) | 47.92 (46.41, 49.80) | 47.17 (45.90, 49.35) | 47.56 (46.05, 49.82) | -0.041 | 0.481 |
| 25-29 years | 48.77 (50.01, 48.34) | 46.66 (49.41, 43.72) | 45.20 (48.40, 45.47) | 46.00 (48.82, 47.31) | 47.02 (47.64, 48.69) | 46.17 (46.49, 47.89) | 46.70 (46.55, 48.54) | -0.087 | 0.396 |
| 30-34 years | 49.13 (50.04, 47.60) | 47.56 (49.95, 44.27) | 45.55 (48.83, 44.29) | 45.89 (49.05, 45.58) | 46.98 (48.36, 47.50) | 46.30 (47.56, 47.15) | 47.22 (47.28, 47.60) | -0.112 | 0.304 |
| 35-39 years | 49.57 (50.44, 47.07) | 48.18 (50.43, 44.47) | 46.47 (49.40, 44.32) | 46.18 (49.44, 44.88) | 47.61 (48.76, 46.30) | 46.91 (48.16, 46.60) | 47.67 (48.25, 47.24) | -0.115 | 0.259 |
| 40-44 years | 49.65 (51.14, 47.35) | 48.16 (50.48, 43.70) | 47.16 (49.86, 44.68) | 46.97 (50.01, 45.24) | 48.03 (49.43, 46.02) | 47.55 (48.46, 46.09) | 48.31 (48.59, 46.94) | -0.072 | 0.374 |
| 45-49 years | 50.18 (51.47, 48.37) | 48.38 (50.53, 45.08) | 46.84 (49.89, 44.66) | 47.57 (50.53, 45.93) | 48.30 (50.00, 46.74) | 47.98 (49.07, 46.31) | 48.94 (49.18, 46.96) | -0.051 | 0.597 |
| 50-54 years | 48.55 (50.26, 48.60) | 48.89 (50.86, 46.38) | 46.99 (49.74, 45.62) | 47.17 (50.45, 46.31) | 48.59 (50.42, 47.76) | 48.14 (49.58, 47.33) | 49.28 (49.67, 47.77) | 0.03 | 0.709 |
| 55-59 years | 48.55 (50.29, 49.68) | 47.54 (49.82, 47.15) | 47.43 (50.01, 46.53) | 47.52 (50.61, 47.52) | 48.92 (50.59, 48.56) | 48.42 (49.97, 48.55) | 49.42 (50.09, 49.04) | 0.087 | 0.196 |
| 60-64 years | 48.96 (49.16, 49.73) | 47.89 (49.70, 48.02) | 47.42 (49.08, 47.80) | 47.86 (50.86, 48.60) | 49.09 (50.89, 49.83) | 48.80 (50.05, 49.48) | 49.86 (50.40, 50.27) | 0.09 | 0.222 |
| 65-69 years | 49.87 (49.38, 50.72) | 48.74 (48.78, 48.83) | 47.80 (48.73, 48.56) | 47.69 (49.97, 50.07) | 49.06 (51.00, 51.05) | 49.10 (50.38, 50.79) | 50.38 (50.71, 51.37) | 0.046 | 0.608 |
| 70-74 years | 50.30 (49.86, 51.19) | 49.69 (49.59, 49.61) | 48.84 (49.05, 50.09) | 48.28 (49.82, 50.92) | 49.66 (50.43, 52.55) | 49.03 (50.45, 51.73) | 50.69 (51.17, 52.51) | 0.003 | 0.972 |
| 75-79 years | 51.88 (51.49, 53.53) | 50.98 (51.17, 53.47) | 53.19 (51.53, 55.24) | 54.49 (51.75, 55.54) | 55.53 (52.75, 56.51) | 54.75 (52.32, 56.59) | 55.12 (52.70, 57.25) | 0.278 | 0.009 |
| 80-84 years | 52.54 (52.57, 52.49) | 52.46 (52.48, 52.77) | 53.94 (52.19, 55.17) | 54.46 (53.35, 55.07) | 54.85 (54.05, 55.25) | 55.54 (53.70, 56.25) | 55.82 (54.16, 56.47) | 0.234 | <0.001 |
| 85-89 years | 51.95 (53.40, 51.39) | 51.80 (53.27, 51.48) | 53.35 (52.96, 53.55) | 54.42 (53.66, 54.73) | 54.47 (53.76, 54.65) | 54.90 (54.26, 55.23) | 55.59 (55.00, 56.07) | 0.254 | 0.001 |
| 90-94 years | 51.45 (52.98, 50.57) | 51.21 (53.04, 50.53) | 52.54 (52.75, 51.90) | 52.73 (52.68, 52.57) | 54.14 (53.78, 54.32) | 54.56 (54.05, 54.50) | 54.76 (54.52, 54.63) | 0.258 | <0.001 |
| 95+ years | 50.58 (52.94, 49.27) | 51.46 (53.98, 50.44) | 52.29 (53.42, 51.89) | 52.46 (52.39, 52.32) | 53.28 (53.07, 53.40) | 54.98 (54.56, 55.15) | 54.81 (54.50, 54.92) | 0.294 | <0.001 |
| >15 years | 49.43 (50.29, 48.99) | 48.19 (49.91, 46.20) | 47.28 (49.27, 46.62) | 47.31 (49.58, 47.61) | 48.46 (49.14, 48.88) | 47.86 (48.39, 48.54) | 48.65 (48.48, 49.12) | -0.032 | 0.655 |
| Rural | 51.20 (50.56, 52.21) | 49.45 (51.94, 47.31) | 48.08 (51.44, 45.79) | 48.02 (51.29, 47.89) | 47.84 (50.86, 48.33) | 47.34 (50.16, 46.77) | 47.53 (50.21, 47.24) | -0.239 | 0.008 |
| Urban | 47.98 (48.20, 47.03) | 47.97 (48.67, 47.18) | 47.61 (48.41, 48.46) | 48.67 (48.94, 49.20) | 50.08 (49.23, 50.17) | 49.63 (48.88, 49.97) | 50.61 (49.81, 50.66) | 0.206 | 0.007 |
| Low (0-6 years formal) | 50.99 (49.19, 53.91) | 52.51 (51.39, 51.55) | 52.24 (52.61, 50.71) | 52.25 (53.63, 52.01) | 51.15 (52.67, 50.36) | 48.25 (50.68, 46.24) | 48.41 (50.72, 46.57) | -0.254 | 0.06 |
| Medium (6.01-12 years) | 48.37 (49.01, 48.34) | 46.14 (48.44, 44.86) | 44.36 (47.19, 45.53) | 45.48 (48.39, 46.77) | 47.13 (48.04, 47.96) | 48.31 (48.16, 49.09) | 49.45 (48.74, 49.90) | 0.154 | 0.356 |
| High (12.01+ years) | 46.15 (48.03, 46.58) | 47.02 (48.99, 47.28) | 48.82 (49.54, 49.46) | 50.31 (49.88, 50.70) | 51.71 (50.91, 51.85) | 51.34 (51.74, 51.04) | 52.11 (52.50, 51.76) | 0.45 | <0.001 |
| **Female** |  |  |  |  |  |  |  |  |  |
| 15-19 years | 50.43 (50.20, 51.05) | 49.87 (49.64, 48.74) | 49.25 (49.44, 49.54) | 47.81 (48.49, 50.25) | 49.48 (48.53, 52.02) | 48.28 (47.54, 51.03) | 48.65 (47.36, 51.45) | -0.128 | 0.076 |
| 20-24 years | 49.36 (50.88, 49.34) | 47.47 (49.91, 46.03) | 47.98 (49.27, 48.34) | 47.88 (49.08, 49.64) | 48.59 (47.52, 50.63) | 47.76 (46.80, 50.09) | 48.14 (46.79, 50.52) | -0.039 | 0.498 |
| 25-29 years | 49.35 (51.15, 48.49) | 47.23 (50.43, 44.28) | 45.91 (49.51, 46.26) | 46.69 (49.78, 48.03) | 47.67 (48.43, 49.39) | 46.81 (47.44, 48.63) | 47.31 (47.47, 49.21) | -0.083 | 0.395 |
| 30-34 years | 49.88 (51.24, 47.90) | 48.23 (51.03, 44.08) | 46.26 (50.04, 44.99) | 46.63 (50.11, 46.29) | 47.65 (49.28, 48.25) | 47.00 (48.44, 47.83) | 47.86 (48.29, 48.33) | -0.115 | 0.283 |
| 35-39 years | 50.44 (51.65, 47.24) | 48.98 (51.45, 44.38) | 47.31 (50.67, 45.03) | 46.98 (50.64, 45.60) | 48.38 (49.99, 47.08) | 47.65 (49.26, 47.37) | 48.40 (49.22, 48.03) | -0.122 | 0.224 |
| 40-44 years | 50.51 (52.20, 47.64) | 48.98 (51.73, 43.60) | 48.06 (51.15, 45.48) | 47.77 (51.32, 46.05) | 48.89 (50.67, 46.84) | 48.33 (49.61, 46.88) | 49.10 (49.84, 47.74) | -0.075 | 0.342 |
| 45-49 years | 50.93 (52.37, 48.42) | 49.35 (51.69, 45.35) | 47.77 (51.13, 45.46) | 48.40 (51.83, 46.84) | 49.23 (51.27, 47.63) | 48.83 (50.26, 47.10) | 49.80 (50.40, 47.80) | -0.049 | 0.592 |
| 50-54 years | 49.31 (51.25, 48.35) | 49.77 (51.97, 46.58) | 48.07 (51.17, 46.57) | 48.13 (51.86, 47.26) | 49.55 (51.75, 48.76) | 49.10 (50.88, 48.29) | 50.21 (50.89, 48.70) | 0.038 | 0.605 |
| 55-59 years | 49.36 (51.14, 49.48) | 48.51 (51.02, 47.53) | 48.47 (51.37, 47.61) | 48.65 (52.03, 48.66) | 49.95 (52.01, 49.67) | 49.44 (51.22, 49.63) | 50.40 (51.37, 50.04) | 0.094 | 0.12 |
| 60-64 years | 49.78 (49.98, 49.61) | 48.99 (50.83, 48.22) | 48.67 (50.46, 49.15) | 48.97 (52.18, 49.81) | 50.26 (52.49, 50.99) | 49.86 (51.39, 50.65) | 50.84 (51.80, 51.41) | 0.094 | 0.141 |
| 65-69 years | 50.53 (50.10, 50.50) | 49.91 (49.91, 49.42) | 49.05 (50.10, 50.18) | 48.99 (51.31, 51.62) | 50.17 (52.34, 52.34) | 50.18 (51.77, 52.05) | 51.40 (51.97, 52.52) | 0.057 | 0.436 |
| 70-74 years | 50.92 (50.53, 51.28) | 50.68 (50.55, 50.19) | 50.04 (50.27, 51.79) | 49.61 (51.22, 52.67) | 51.05 (51.59, 54.11) | 50.26 (51.68, 53.36) | 51.78 (52.39, 54.03) | 0.035 | 0.586 |
| 75-79 years | 52.57 (52.22, 53.10) | 52.05 (52.24, 54.43) | 54.76 (52.71, 55.29) | 55.31 (53.08, 55.33) | 55.88 (53.97, 56.10) | 55.94 (53.14, 56.29) | 56.27 (53.56, 57.37) | 0.278 | 0.004 |
| 80-84 years | 52.75 (53.29, 51.97) | 52.65 (53.46, 52.26) | 54.70 (53.25, 55.41) | 54.66 (54.11, 54.36) | 55.01 (54.43, 54.18) | 55.70 (54.54, 56.29) | 55.97 (54.99, 56.25) | 0.222 | 0.001 |
| 85-89 years | 52.16 (53.64, 50.98) | 51.94 (53.61, 50.90) | 53.35 (53.30, 52.54) | 54.15 (53.83, 54.25) | 54.21 (54.01, 53.33) | 54.84 (54.53, 54.40) | 55.34 (55.16, 55.43) | 0.226 | <0.001 |
| 90-94 years | 51.67 (53.33, 50.18) | 51.37 (53.45, 49.86) | 52.31 (53.09, 51.05) | 52.51 (52.49, 51.70) | 53.94 (53.66, 53.60) | 54.20 (54.19, 53.83) | 54.26 (54.30, 53.91) | 0.213 | 0.001 |
| 95+ years | 50.82 (53.60, 49.12) | 51.66 (54.48, 49.89) | 52.16 (53.70, 51.36) | 52.33 (52.45, 51.83) | 53.19 (52.99, 52.85) | 54.63 (54.51, 54.77) | 54.45 (54.39, 54.56) | 0.253 | <0.001 |
| >15 years | 50.14 (51.24, 49.09) | 48.99 (50.96, 46.44) | 48.19 (50.45, 47.52) | 48.18 (50.74, 48.52) | 49.31 (50.30, 49.75) | 48.71 (49.48, 49.41) | 49.46 (49.56, 49.98) | -0.026 | 0.691 |
| Rural | 51.93 (51.49, 52.44) | 49.99 (53.01, 46.77) | 48.78 (52.52, 46.50) | 48.70 (52.43, 48.29) | 48.43 (51.90, 48.80) | 47.86 (51.12, 47.57) | 47.99 (51.13, 48.05) | -0.25 | 0.005 |
| Urban | 48.81 (49.40, 47.78) | 48.91 (50.05, 48.07) | 48.62 (49.98, 49.40) | 49.77 (50.56, 50.11) | 51.14 (50.44, 50.97) | 50.66 (49.86, 50.90) | 51.61 (50.73, 51.59) | 0.214 | 0.004 |
| Low (0-6 years formal) | 51.88 (49.96, 53.94) | 53.01 (51.81, 50.38) | 52.64 (52.96, 49.98) | 52.49 (53.77, 50.94) | 51.28 (53.12, 49.78) | 48.68 (51.51, 46.79) | 48.84 (51.55, 47.16) | -0.278 | 0.026 |
| Medium (6.01-12 years) | 49.26 (50.30, 47.95) | 47.23 (49.87, 45.38) | 45.59 (48.97, 46.16) | 46.53 (50.18, 47.17) | 48.09 (49.94, 48.39) | 49.30 (49.84, 49.78) | 50.40 (50.10, 50.52) | 0.147 | 0.342 |
| High (12.01+ years) | 47.26 (49.78, 47.35) | 48.22 (50.93, 48.05) | 49.97 (51.44, 50.08) | 51.42 (51.66, 51.34) | 52.65 (52.03, 52.37) | 52.46 (52.86, 51.41) | 53.18 (53.63, 52.16) | 0.433 | <0.001 |
| **Male** |  |  |  |  |  |  |  |  |  |
| 15-19 years | 49.40 (48.90, 50.06) | 48.87 (49.03, 48.09) | 48.31 (48.37, 48.16) | 46.77 (47.74, 48.71) | 48.19 (47.57, 50.65) | 47.12 (46.56, 49.73) | 47.52 (46.41, 50.22) | -0.146 | 0.044 |
| 20-24 years | 48.28 (49.32, 49.15) | 46.39 (49.02, 44.61) | 46.93 (48.38, 47.02) | 46.85 (48.29, 48.20) | 47.25 (46.71, 49.13) | 46.57 (45.83, 48.74) | 47.00 (46.15, 49.27) | -0.051 | 0.365 |
| 25-29 years | 48.23 (49.25, 48.16) | 46.46 (49.46, 43.59) | 44.88 (48.37, 44.76) | 45.63 (48.72, 46.65) | 46.37 (47.70, 48.08) | 45.54 (46.28, 47.24) | 46.11 (46.50, 47.96) | -0.11 | 0.251 |
| 30-34 years | 48.33 (49.28, 47.70) | 47.19 (49.86, 43.67) | 45.23 (48.78, 43.90) | 45.31 (48.96, 45.57) | 46.43 (48.32, 46.97) | 45.71 (47.37, 46.57) | 46.65 (47.09, 46.98) | -0.113 | 0.262 |
| 35-39 years | 48.67 (49.61, 47.68) | 47.68 (50.02, 43.87) | 45.90 (49.21, 43.80) | 45.67 (49.42, 45.29) | 46.98 (48.66, 46.70) | 46.27 (48.05, 45.93) | 47.02 (48.05, 46.59) | -0.11 | 0.244 |
| 40-44 years | 48.94 (50.11, 47.68) | 47.64 (50.11, 43.86) | 46.55 (49.45, 43.91) | 46.45 (49.88, 45.14) | 47.37 (49.36, 46.69) | 46.89 (48.22, 45.35) | 47.59 (48.45, 46.22) | -0.078 | 0.308 |
| 45-49 years | 49.57 (50.82, 49.03) | 47.63 (49.97, 44.80) | 46.18 (49.48, 44.44) | 46.96 (50.18, 45.61) | 47.54 (49.88, 46.96) | 47.22 (48.81, 45.87) | 48.21 (48.73, 46.13) | -0.06 | 0.547 |
| 50-54 years | 48.00 (49.90, 49.09) | 48.26 (50.52, 46.33) | 46.21 (49.33, 45.11) | 46.55 (50.07, 46.26) | 47.77 (50.06, 47.93) | 47.34 (49.26, 46.44) | 48.50 (49.50, 46.82) | 0.014 | 0.869 |
| 55-59 years | 47.96 (49.78, 50.24) | 46.86 (49.39, 46.85) | 46.57 (49.54, 46.28) | 46.68 (50.05, 46.91) | 48.05 (50.21, 49.05) | 47.55 (49.69, 47.50) | 48.57 (49.70, 48.05) | 0.069 | 0.323 |
| 60-64 years | 48.08 (48.94, 50.15) | 46.78 (49.14, 47.72) | 46.16 (48.56, 47.39) | 47.05 (50.34, 48.20) | 48.06 (50.46, 49.81) | 47.86 (49.69, 48.42) | 48.93 (50.02, 49.11) | 0.099 | 0.235 |
| 65-69 years | 49.12 (49.25, 51.22) | 47.50 (48.38, 48.59) | 46.48 (48.16, 48.23) | 46.35 (49.44, 49.13) | 47.99 (50.57, 51.13) | 48.03 (49.99, 49.63) | 49.39 (50.48, 50.33) | 0.045 | 0.685 |
| 70-74 years | 49.53 (50.19, 51.77) | 48.44 (48.55, 48.90) | 47.37 (47.94, 48.87) | 46.81 (49.22, 49.99) | 47.96 (50.08, 52.09) | 47.93 (50.04, 50.36) | 49.58 (50.66, 51.15) | -0.013 | 0.893 |
| 75-79 years | 50.89 (50.56, 52.77) | 49.64 (50.01, 52.31) | 50.95 (50.18, 54.02) | 52.15 (49.73, 55.17) | 53.41 (50.56, 57.58) | 53.12 (50.30, 56.19) | 53.63 (51.10, 56.61) | 0.255 | 0.006 |
| 80-84 years | 51.71 (51.30, 53.03) | 50.96 (51.06, 53.02) | 51.53 (50.87, 54.70) | 53.41 (51.22, 54.89) | 54.26 (51.99, 55.80) | 54.30 (51.46, 56.27) | 55.03 (52.18, 56.54) | 0.273 | 0.003 |
| 85-89 years | 51.75 (52.48, 52.01) | 51.58 (52.63, 52.00) | 52.82 (52.03, 53.55) | 53.69 (52.09, 54.76) | 53.76 (52.78, 54.81) | 54.23 (52.89, 55.23) | 55.17 (53.47, 56.12) | 0.23 | <0.001 |
| 90-94 years | 50.86 (52.60, 51.07) | 50.75 (52.77, 51.12) | 52.14 (52.36, 52.67) | 52.74 (52.00, 53.49) | 53.57 (52.60, 54.63) | 53.63 (52.74, 54.58) | 54.29 (53.30, 55.27) | 0.249 | <0.001 |
| 95+ years | 49.79 (52.04, 49.31) | 50.83 (53.09, 51.07) | 51.95 (52.61, 52.65) | 52.73 (52.00, 53.18) | 53.11 (52.23, 53.99) | 54.00 (52.96, 55.23) | 54.40 (53.32, 55.59) | 0.306 | <0.001 |
| >15 years | 48.75 (49.63, 49.14) | 47.55 (49.60, 45.71) | 46.55 (48.91, 46.00) | 46.60 (49.27, 47.19) | 47.63 (48.89, 48.74) | 47.06 (48.01, 47.77) | 47.87 (48.14, 48.33) | -0.044 | 0.544 |
| Rural | 50.70 (49.20, 52.68) | 49.30 (51.73, 47.49) | 47.95 (51.56, 46.98) | 47.85 (51.68, 48.70) | 47.72 (51.33, 49.59) | 47.21 (50.47, 47.63) | 47.38 (50.55, 47.75) | -0.223 | 0.006 |
| Urban | 47.16 (47.38, 46.07) | 47.05 (47.92, 46.31) | 46.59 (47.70, 47.53) | 47.61 (48.39, 48.19) | 49.07 (48.45, 49.30) | 48.68 (47.92, 49.01) | 49.66 (48.77, 49.73) | 0.203 | 0.011 |
| Low (0-6 years formal) | 50.14 (48.03, 54.51) | 52.26 (50.16, 51.95) | 52.04 (51.33, 51.77) | 52.37 (52.71, 52.60) | 51.28 (53.09, 51.73) | 48.20 (51.05, 47.22) | 48.32 (51.02, 47.13) | -0.209 | 0.152 |
| Medium (6.01-12 years) | 47.52 (48.24, 48.33) | 45.50 (48.21, 45.01) | 43.49 (47.08, 45.43) | 44.71 (48.68, 46.98) | 46.42 (48.50, 48.27) | 47.45 (48.11, 48.49) | 48.64 (48.46, 49.23) | 0.154 | 0.362 |
| High (12.01+ years) | 45.04 (47.35, 45.71) | 45.89 (48.51, 46.29) | 47.70 (49.15, 48.46) | 49.19 (49.41, 49.79) | 50.75 (50.01, 51.10) | 50.23 (50.57, 50.05) | 51.04 (51.37, 50.84) | 0.465 | <0.001 |

**Supplementary Table 4** Distribution of PHDI scores among populations aged 15 years and older in 185 countries, 2018

| **Country** | **Total** | **Female** | **Male** |
| --- | --- | --- | --- |
| Antigua and Barbuda | 46.36 | 46.67 | 46.4 |
| Arab Republic of Egypt | 32.85 | 34.09 | 31.86 |
| Argentine Republic | 41.98 | 42.16 | 41.76 |
| Australia | 47.51 | 48.52 | 47.31 |
| Barbados | 42.25 | 43.55 | 42.09 |
| Belize | 45.48 | 45.94 | 45.02 |
| Bolivarian Republic of Venezuela | 43.33 | 43.47 | 43.81 |
| Bosnia and Herzegovina | 41.43 | 41.95 | 41.31 |
| Brunei Darussalam | 38.27 | 39.15 | 36.03 |
| Burkina Faso | 54.31 | 54.34 | 55.32 |
| Canada | 57.16 | 58.31 | 55.71 |
| Central African Republic | 56.17 | 55.47 | 55.23 |
| Commonwealth of Dominica | 47.9 | 47.98 | 46.98 |
| Commonwealth of the Bahamas | 48.22 | 48.55 | 48.36 |
| Czech Republic | 42.06 | 43.17 | 40.82 |
| Democratic Republic of Sao Tome and Principe | 43 | 43.17 | 43.23 |
| Democratic Republic of Timor-Leste | 40.51 | 41.1 | 40.3 |
| Democratic Republic of the Congo | 48.7 | 49.28 | 49.35 |
| Democratic Socialist Republic of Sri Lanka | 69.01 | 68.47 | 69.32 |
| Dominican Republic | 46.52 | 45.83 | 46.86 |
| Eastern Republic of Uruguay | 59.54 | 60 | 58.88 |
| Federal Democratic Republic of Ethiopia | 45.08 | 46.14 | 45.88 |
| Federal Democratic Republic of Nepal | 54.95 | 53.75 | 56.87 |
| Federal Republic of Germany | 49.96 | 50.37 | 48.82 |
| Federal Republic of Nigeria | 50.95 | 51.68 | 50.97 |
| Federated States of Micronesia | 41.03 | 41.68 | 38.64 |
| Federative Republic of Brazil | 57.82 | 59.59 | 55.99 |
| French Republic | 60.42 | 59.76 | 59.29 |
| Gabonese Republic | 52.82 | 52.67 | 53.01 |
| Georgia | 36.31 | 36.61 | 35.35 |
| Grand Duchy of Luxembourg | 39.09 | 39.94 | 38.27 |
| Grenada | 47.66 | 48.24 | 47.59 |
| Hashemite Kingdom of Jordan | 51.07 | 50.06 | 51.83 |
| Hellenic Republic | 44.91 | 46.31 | 44 |
| Hungary | 42.96 | 44.03 | 42.41 |
| Independent State of Papua New Guinea | 32.86 | 33.03 | 32.16 |
| Independent State of Samoa | 68.25 | 68.46 | 66.33 |
| Ireland | 38.08 | 39.26 | 37.33 |
| Islamic Republic of Afghanistan | 62.49 | 62.12 | 61.73 |
| Islamic Republic of Iran | 62.55 | 63.28 | 61.6 |
| Islamic Republic of Mauritania | 54.27 | 54.69 | 53.98 |
| Islamic Republic of Pakistan | 34.96 | 34.72 | 35.43 |
| Jamaica | 46.35 | 47.06 | 46.71 |
| Japan | 57.98 | 58.85 | 56.63 |
| Kingdom of Bahrain | 52.96 | 54.36 | 51.64 |
| Kingdom of Belgium | 53.77 | 54.96 | 51.94 |
| Kingdom of Bhutan | 58.09 | 57.4 | 58.57 |
| Kingdom of Cambodia | 33.17 | 34.38 | 33.86 |
| Kingdom of Denmark | 49.02 | 51.79 | 48.48 |
| Kingdom of Eswatini | 36.66 | 37.77 | 35.91 |
| Kingdom of Lesotho | 45.38 | 46.1 | 44.03 |
| Kingdom of Morocco | 49.84 | 50.88 | 47.39 |
| Kingdom of Norway | 32.87 | 35.48 | 32.52 |
| Kingdom of Saudi Arabia | 54.67 | 54.99 | 52.61 |
| Kingdom of Spain | 32.74 | 33.87 | 32.58 |
| Kingdom of Sweden | 30.45 | 32.27 | 29.43 |
| Kingdom of Thailand | 47.18 | 48.44 | 45.15 |
| Kingdom of Tonga | 46.61 | 47.11 | 44.38 |
| Kingdom of the Netherlands | 57.19 | 57.6 | 57.14 |
| Kyrgyz Republic | 31.74 | 32.58 | 30.31 |
| Lao People's Democratic Republic | 31.52 | 33.16 | 32.35 |
| Lebanese Republic | 57.2 | 62.76 | 54.35 |
| Malaysia | 47.71 | 49.04 | 47.45 |
| Mongolia | 50.22 | 51.24 | 49.64 |
| Montenegro | 46.16 | 45.96 | 46.28 |
| New Zealand | 50.66 | 52.23 | 50.03 |
| North Macedonia | 49.07 | 50.44 | 48.06 |
| People's Democratic Republic of Algeria | 45.7 | 46.51 | 44.52 |
| People's Republic of Bangladesh | 60.84 | 60.76 | 60.94 |
| People's Republic of China | 55.97 | 56.59 | 55.12 |
| Plurinational State of Bolivia | 39.74 | 39.84 | 39.1 |
| Portuguese Republic | 43.03 | 42.81 | 43.77 |
| Republic of Albania | 52.17 | 52.92 | 50.59 |
| Republic of Angola | 56.46 | 55.94 | 56.94 |
| Republic of Armenia | 35.85 | 36.91 | 34.15 |
| Republic of Austria | 43.45 | 44.95 | 41.66 |
| Republic of Azerbaijan | 50.41 | 51.6 | 48.77 |
| Republic of Belarus | 53.96 | 54.75 | 53.48 |
| Republic of Benin | 55.34 | 55.73 | 54.31 |
| Republic of Botswana | 60.09 | 60.73 | 59.82 |
| Republic of Bulgaria | 54.9 | 55.64 | 54.01 |
| Republic of Burundi | 54.78 | 55.8 | 55.86 |
| Republic of Cabo Verde | 67.13 | 67.96 | 67.72 |
| Republic of Cameroon | 59.6 | 59.96 | 60.2 |
| Republic of Chad | 51.16 | 51.04 | 51.36 |
| Republic of Chile | 47.96 | 48.05 | 46.69 |
| Republic of Colombia | 46.9 | 47.03 | 46.5 |
| Republic of Costa Rica | 44.5 | 45.08 | 44.25 |
| Republic of Croatia | 38.76 | 38.91 | 38.9 |
| Republic of Cuba | 43 | 43.52 | 42.68 |
| Republic of Cyprus | 51.58 | 52.88 | 50.39 |
| Republic of Côte d’Ivoire | 46.5 | 47.17 | 47.53 |
| Republic of Djibouti | 49.38 | 49.04 | 49 |
| Republic of Ecuador | 39.59 | 40.3 | 38.72 |
| Republic of El Salvador | 43.17 | 43.02 | 41.88 |
| Republic of Equatorial Guinea | 50.43 | 50.35 | 49.96 |
| Republic of Estonia | 49.53 | 51.13 | 47.96 |
| Republic of Fiji | 52.74 | 53.57 | 51.65 |
| Republic of Finland | 40.79 | 42.68 | 38.7 |
| Republic of Ghana | 55.01 | 54.13 | 56.55 |
| Republic of Guatemala | 37.93 | 38.1 | 37.88 |
| Republic of Guinea | 41.5 | 41.63 | 40.9 |
| Republic of Guinea-Bissau | 55.54 | 55.58 | 55.73 |
| Republic of Guyana | 44.42 | 44.5 | 44.44 |
| Republic of Haiti | 48.96 | 48.69 | 49.56 |
| Republic of Honduras | 38.67 | 39.01 | 38.51 |
| Republic of Iceland | 26.95 | 28.43 | 26.35 |
| Republic of India | 54.27 | 56.35 | 51.85 |
| Republic of Indonesia | 36.58 | 36.7 | 36.1 |
| Republic of Iraq | 53.26 | 54.61 | 52.1 |
| Republic of Italy | 49.4 | 50.09 | 48.79 |
| Republic of Kazakhstan | 49.59 | 51.2 | 47.99 |
| Republic of Kenya | 56.28 | 56.76 | 56.89 |
| Republic of Kiribati | 36.28 | 36.89 | 34.24 |
| Republic of Korea | 46.92 | 49.19 | 44.52 |
| Republic of Latvia | 42.45 | 42.84 | 42.71 |
| Republic of Liberia | 45.56 | 45.6 | 46.93 |
| Republic of Lithuania | 33.62 | 34.47 | 32.2 |
| Republic of Madagascar | 47.94 | 48.42 | 48.12 |
| Republic of Malawi | 56.43 | 57.39 | 56.19 |
| Republic of Maldives | 62.44 | 59.75 | 63.91 |
| Republic of Mali | 53.71 | 54.24 | 53.69 |
| Republic of Malta | 52.85 | 55.03 | 51.53 |
| Republic of Mauritius | 50.31 | 51.09 | 50.03 |
| Republic of Moldova | 57.35 | 58.58 | 55.95 |
| Republic of Mozambique | 49.75 | 49.74 | 51.21 |
| Republic of Namibia | 56.07 | 56.12 | 56.26 |
| Republic of Nicaragua | 52.49 | 52.28 | 51.6 |
| Republic of Panama | 65.33 | 65.86 | 65.05 |
| Republic of Paraguay | 35.48 | 35.61 | 34.29 |
| Republic of Peru | 42.51 | 43.08 | 41.89 |
| Republic of Poland | 43.18 | 44.33 | 41.75 |
| Republic of Rwanda | 52.29 | 52.78 | 51.21 |
| Republic of Senegal | 37.29 | 37.87 | 36.97 |
| Republic of Serbia | 67.26 | 67.56 | 66.26 |
| Republic of Seychelles | 48.27 | 49.9 | 47.39 |
| Republic of Sierra Leone | 55.4 | 55.57 | 56.82 |
| Republic of Singapore | 41.66 | 42.74 | 39.26 |
| Republic of Slovenia | 41.36 | 42 | 41.1 |
| Republic of South Africa | 36.75 | 37.26 | 36.79 |
| Republic of South Sudan | 48.19 | 48.94 | 48.83 |
| Republic of Suriname | 58.97 | 59.82 | 58.95 |
| Republic of Tajikistan | 36.46 | 37.56 | 34.82 |
| Republic of Trinidad and Tobago | 49.51 | 50.18 | 49.62 |
| Republic of Tunisia | 40.81 | 42.02 | 39.45 |
| Republic of Turkey | 45.96 | 47.36 | 44.81 |
| Republic of Uganda | 56.66 | 56.72 | 57.53 |
| Republic of Uzbekistan | 44.43 | 46.31 | 43.49 |
| Republic of Vanuatu | 42.57 | 42.26 | 45.48 |
| Republic of Yemen | 41.58 | 42.5 | 40.72 |
| Republic of Zambia | 66.54 | 65.82 | 67.14 |
| Republic of Zimbabwe | 57.56 | 57.42 | 57.39 |
| Republic of the Congo | 41.32 | 41.72 | 41.69 |
| Republic of the Gambia | 41.75 | 41.16 | 41.74 |
| Republic of the Marshall Islands | 48.16 | 48.9 | 45.83 |
| Republic of the Niger | 58.66 | 58.5 | 57.02 |
| Republic of the Philippines | 46.79 | 47.53 | 46.04 |
| Republic of the Sudan | 53.58 | 54.97 | 53.96 |
| Republic of the Union of Myanmar | 39.78 | 40.3 | 38.49 |
| Romania | 47.03 | 46.2 | 47.92 |
| Russian Federation | 43.31 | 44.55 | 42.55 |
| Saint Lucia | 51.1 | 51.36 | 50.38 |
| Saint Vincent and the Grenadines | 58.19 | 58.57 | 58.3 |
| Slovak Republic | 45.54 | 47.24 | 43.74 |
| Socialist Republic of Viet Nam | 40.73 | 40.21 | 41.37 |
| Solomon Islands | 62.76 | 63.52 | 62.38 |
| State of Eritrea | 42.06 | 42.47 | 41.84 |
| State of Israel | 43.32 | 45.66 | 41.08 |
| State of Kuwait | 38.77 | 37.75 | 38.32 |
| State of Libya | 46.19 | 46.55 | 45.18 |
| State of Palestine | 51.8 | 51.66 | 50.62 |
| State of Qatar | 52.59 | 54.73 | 51.96 |
| Sultanate of Oman | 53.13 | 54.14 | 51.93 |
| Swiss Confederation | 46.19 | 46.47 | 45.69 |
| Syrian Arab Republic | 51.72 | 51.47 | 50.51 |
| Taiwan (Province of China) | 49.81 | 50.46 | 48.47 |
| Togolese Republic | 44 | 44.33 | 43.66 |
| Turkmenistan | 56.1 | 56.84 | 54.65 |
| Ukraine | 45.92 | 46.85 | 44.38 |
| Union of the Comoros | 43.61 | 43.86 | 44.07 |
| United Arab Emirates | 54.66 | 54.19 | 53.63 |
| United Kingdom of Great Britain and Northern Ireland | 46.05 | 47.64 | 45.66 |
| United Mexican States | 47.11 | 47.47 | 46.58 |
| United Republic of Tanzania | 65.11 | 64.99 | 66.62 |
| United States of America | 52.43 | 53.24 | 51.99 |

**Supplementary Table 5** PHDI component scores derived from the GDD in 2018

|  | **saturated oils** | **unsaturated oils** | **nonstarchy veg** | **eggs** | **legumes** | **starchy veg** | **grains** | **red meat** | **nuts seeds** | **dairy** | **fruits** | **sugar** | **fish** |
| --- | --- | --- | --- | --- | --- | --- | --- | --- | --- | --- | --- | --- | --- |
| **Total** |  |  |  |  |  |  |  |  |  |  |  |  |  |
| 15-19 years | 0 | 9.15 | 7.67 | 0 | 5.75 | 0 | 3.05 | 0 | 2.03 | 6.76 | 5.17 | 0 | 8.5 |
| 20-24 years | 0 | 9.28 | 8.09 | 0 | 5.4 | 0 | 2.97 | 0 | 2.11 | 6.07 | 5.14 | 0 | 8.52 |
| 25-29 years | 0 | 9.42 | 8.42 | 0 | 4.78 | 0 | 2.79 | 0 | 2.2 | 5.42 | 5.06 | 0 | 8.6 |
| 30-34 years | 0 | 9.5 | 8.61 | 0 | 4.37 | 1.21 | 2.68 | 0 | 2.22 | 4.98 | 5.01 | 0 | 8.63 |
| 35-39 years | 0 | 9.53 | 8.46 | 0 | 4.25 | 1.97 | 2.71 | 0 | 2.18 | 4.87 | 5.01 | 0 | 8.67 |
| 40-44 years | 0 | 9.45 | 8.72 | 0 | 4.12 | 2.92 | 2.58 | 0 | 2.16 | 4.73 | 5.1 | 0 | 8.52 |
| 45-49 years | 0 | 9.28 | 9.08 | 0 | 4.05 | 3.7 | 2.45 | 0 | 2.16 | 4.66 | 5.26 | 0 | 8.3 |
| 50-54 years | 0 | 9.15 | 9.15 | 0 | 4.15 | 3.54 | 2.48 | 0 | 2.16 | 4.96 | 5.5 | 0 | 8.19 |
| 55-59 years | 0 | 9.11 | 9.01 | 0 | 4.37 | 2.64 | 2.64 | 0 | 2.15 | 5.58 | 5.79 | 0 | 8.13 |
| 60-64 years | 0 | 8.97 | 9.13 | 0 | 4.55 | 2.22 | 2.7 | 0 | 2.14 | 6.06 | 6.09 | 0 | 7.99 |
| 65-69 years | 0 | 8.85 | 9.29 | 0 | 4.75 | 1.91 | 2.69 | 0 | 2.14 | 6.51 | 6.44 | 0 | 7.8 |
| 70-74 years | 0 | 8.85 | 9.06 | 0 | 5.1 | 1.03 | 2.77 | 0 | 2.07 | 7.31 | 6.82 | 0 | 7.69 |
| 75-79 years | 0 | 8.82 | 10 | 0 | 6.25 | 0 | 3.39 | 0 | 2.39 | 9.41 | 8.45 | 0 | 6.41 |
| 80-84 years | 0 | 8.87 | 10 | 0 | 6.4 | 0 | 3.45 | 0 | 2.35 | 9.52 | 9.08 | 0 | 6.13 |
| 85-89 years | 0 | 8.89 | 10 | 0 | 6.83 | 0 | 3.32 | 0 | 2.33 | 8.53 | 9.75 | 0 | 5.94 |
| 90-94 years | 0 | 9.08 | 10 | 0 | 7.01 | 0 | 3.38 | 0 | 2.28 | 7.15 | 10 | 0 | 5.86 |
| 95+ years | 0 | 8.97 | 10 | 0 | 7.74 | 0 | 3.24 | 0 | 2.35 | 6.68 | 10 | 0 | 5.84 |
| >15 years | 0 | 9.25 | 8.67 | 0 | 4.74 | 1.54 | 2.76 | 0 | 2.16 | 5.71 | 5.53 | 0 | 8.29 |
| **Female** |  |  |  |  |  |  |  |  |  |  |  |  |  |
| 15-19 years | 0 | 9.13 | 7.92 | 0 | 5.82 | 0 | 3.02 | 0 | 2.04 | 6.99 | 5.46 | 0 | 8.27 |
| 20-24 years | 0 | 9.25 | 8.37 | 0 | 5.46 | 0 | 2.93 | 0 | 2.12 | 6.27 | 5.44 | 0 | 8.31 |
| 25-29 years | 0 | 9.38 | 8.74 | 0 | 4.83 | 0 | 2.76 | 0 | 2.2 | 5.58 | 5.39 | 0 | 8.43 |
| 30-34 years | 0 | 9.45 | 8.95 | 0 | 4.4 | 1.24 | 2.65 | 0 | 2.21 | 5.13 | 5.35 | 0 | 8.47 |
| 35-39 years | 0 | 9.5 | 8.81 | 0 | 4.28 | 2.06 | 2.67 | 0 | 2.17 | 5.03 | 5.35 | 0 | 8.53 |
| 40-44 years | 0 | 9.43 | 9.08 | 0 | 4.15 | 2.97 | 2.56 | 0 | 2.15 | 4.89 | 5.46 | 0 | 8.39 |
| 45-49 years | 0 | 9.27 | 9.45 | 0 | 4.09 | 3.73 | 2.43 | 0 | 2.15 | 4.83 | 5.66 | 0 | 8.18 |
| 50-54 years | 0 | 9.17 | 9.51 | 0 | 4.2 | 3.56 | 2.47 | 0 | 2.16 | 5.14 | 5.94 | 0 | 8.07 |
| 55-59 years | 0 | 9.13 | 9.37 | 0 | 4.42 | 2.63 | 2.64 | 0 | 2.15 | 5.81 | 6.24 | 0 | 8 |
| 60-64 years | 0 | 9.01 | 9.48 | 0 | 4.61 | 2.1 | 2.71 | 0 | 2.15 | 6.33 | 6.58 | 0 | 7.87 |
| 65-69 years | 0 | 8.92 | 9.65 | 0 | 4.81 | 1.7 | 2.69 | 0 | 2.15 | 6.83 | 6.97 | 0 | 7.68 |
| 70-74 years | 0 | 8.93 | 9.41 | 0 | 5.17 | 0.85 | 2.79 | 0 | 2.07 | 7.65 | 7.33 | 0 | 7.58 |
| 75-79 years | 0 | 8.91 | 10 | 0 | 6.33 | 0 | 3.43 | 0 | 2.38 | 9.89 | 9.08 | 0 | 6.27 |
| 80-84 years | 0 | 8.96 | 10 | 0 | 6.47 | 0 | 3.49 | 0 | 2.34 | 9.01 | 9.72 | 0 | 5.97 |
| 85-89 years | 0 | 9 | 10 | 0 | 6.91 | 0 | 3.31 | 0 | 2.31 | 8.04 | 10 | 0 | 5.77 |
| 90-94 years | 0 | 9.18 | 10 | 0 | 7.06 | 0 | 3.37 | 0 | 2.24 | 6.7 | 10 | 0 | 5.72 |
| 95+ years | 0 | 9.05 | 10 | 0 | 7.83 | 0 | 3.2 | 0 | 2.31 | 6.37 | 10 | 0 | 5.69 |
| >15 years | 0 | 9.24 | 9 | 0 | 4.81 | 1.53 | 2.74 | 0 | 2.16 | 5.92 | 5.95 | 0 | 8.11 |
| **Male** |  |  |  |  |  |  |  |  |  |  |  |  |  |
| 15-19 years | 0 | 9.17 | 7.44 | 0 | 5.67 | 0 | 3.08 | 0 | 2.02 | 6.54 | 4.9 | 0 | 8.72 |
| 20-24 years | 0 | 9.29 | 7.82 | 0 | 5.33 | 0 | 3 | 0 | 2.1 | 5.88 | 4.85 | 0 | 8.72 |
| 25-29 years | 0 | 9.45 | 8.12 | 0 | 4.74 | 0 | 2.82 | 0 | 2.2 | 5.26 | 4.76 | 0 | 8.78 |
| 30-34 years | 0 | 9.51 | 8.27 | 0 | 4.33 | 1.29 | 2.71 | 0 | 2.23 | 4.83 | 4.69 | 0 | 8.79 |
| 35-39 years | 0 | 9.55 | 8.12 | 0 | 4.21 | 1.99 | 2.74 | 0 | 2.19 | 4.71 | 4.68 | 0 | 8.82 |
| 40-44 years | 0 | 9.46 | 8.37 | 0 | 4.08 | 2.95 | 2.61 | 0 | 2.17 | 4.57 | 4.74 | 0 | 8.65 |
| 45-49 years | 0 | 9.27 | 8.7 | 0 | 4 | 3.85 | 2.46 | 0 | 2.16 | 4.49 | 4.85 | 0 | 8.43 |
| 50-54 years | 0 | 9.11 | 8.76 | 0 | 4.1 | 3.72 | 2.49 | 0 | 2.16 | 4.77 | 5.06 | 0 | 8.32 |
| 55-59 years | 0 | 9.07 | 8.64 | 0 | 4.32 | 2.83 | 2.65 | 0 | 2.15 | 5.34 | 5.32 | 0 | 8.26 |
| 60-64 years | 0 | 8.9 | 8.74 | 0 | 4.49 | 2.5 | 2.7 | 0 | 2.13 | 5.75 | 5.57 | 0 | 8.13 |
| 65-69 years | 0 | 8.76 | 8.9 | 0 | 4.68 | 2.29 | 2.67 | 0 | 2.13 | 6.15 | 5.87 | 0 | 7.93 |
| 70-74 years | 0 | 8.77 | 8.65 | 0 | 5.01 | 1.4 | 2.75 | 0 | 2.07 | 6.9 | 6.21 | 0 | 7.82 |
| 75-79 years | 0 | 8.7 | 10 | 0 | 6.16 | 0 | 3.34 | 0 | 2.4 | 8.77 | 7.66 | 0 | 6.6 |
| 80-84 years | 0 | 8.72 | 10 | 0 | 6.3 | 0 | 3.4 | 0 | 2.37 | 9.71 | 8.17 | 0 | 6.36 |
| 85-89 years | 0 | 8.71 | 9.86 | 0 | 6.69 | 0 | 3.34 | 0 | 2.36 | 9.37 | 8.64 | 0 | 6.2 |
| 90-94 years | 0 | 8.85 | 9.44 | 0 | 6.9 | 0 | 3.39 | 0 | 2.35 | 8.15 | 9.05 | 0 | 6.17 |
| 95+ years | 0 | 8.72 | 9.2 | 0 | 7.49 | 0 | 3.35 | 0 | 2.44 | 7.55 | 9.41 | 0 | 6.25 |
| >15 years | 0 | 9.24 | 8.33 | 0 | 4.68 | 1.63 | 2.77 | 0 | 2.16 | 5.48 | 5.1 | 0 | 8.47 |

**Supplementary Table 6** Characteristics of participants in the NHANES

| **Variables** | **Total** | **non-MASLD** | **MASLD** | ***P*** |
| --- | --- | --- | --- | --- |
| Number, weighted | 76190346.93 | 30591194.20 | 45599152.73 |  |
| Age, years, Mean (SD) | 49.67 (16.33) | 45.12 (16.22) | 52.71 (15.68) | <0.001 |
| Sex, n (unweighted) (%) |  |  |  | <0.001 |
| Male | 3626 (45.78) | 1587(54.53) | 2039(39.91) |  |
| Female | 4132 (54.22) | 1370 (45.47) | 2762 (60.09) |  |
| Race, n (unweighted) (%) |  |  |  | <0.001 |
| Mexican American | 1160 ( 5.91) | 221 ( 3.28) | 939 ( 7.67) |  |
| Other Hispanic | 535 ( 4.28) | 168 ( 3.77) | 367 ( 4.63) |  |
| Non-Hispanic White | 3963 (74.05) | 1468 (72.17) | 2495 (75.32) |  |
| Non-Hispanic Black | 1411 ( 9.44) | 789 (13.92) | 622 ( 6.44) |  |
| Other Race | 689 ( 6.31) | 311 ( 6.86) | 378 ( 5.95) |  |
| Education, n (unweighted) (%) |  |  |  | <0.001 |
| Less than high school | 712 ( 4.37) | 172 ( 3.31) | 540 ( 5.09) |  |
| High school or equivalent | 2671 (31.75) | 917 (27.36) | 1754 (34.70) |  |
| Above high school | 4375 (63.87) | 1868 (69.33) | 2507 (60.21) |  |
| Marry, n (unweighted) (%) |  |  |  | 0.006 |
| Living alone | 2559 (28.38) | 1069 (31.32) | 1490 (26.41) |  |
| Married or living with a partner | 5199 (71.62) | 1888 (68.68) | 3311 (73.59) |  |
| PIR, n (unweighted) (%) |  |  |  | 0.060 |
| Low | 1963 (16.06) | 718(16.25) | 1245 (15.93) |  |
| Medium | 2954 (35.50) | 1058 (33.30) | 1896 (36.98) |  |
| High | 2841(48.44) | 1181 (50.44) | 1660 (47.09) |  |
| BMI, kg/m2, Mean (SD) | 28.80 (6.457) | 24.59 (3.981) | 31.62 (6.259) | <0.001 |
| Smoking, n (unweighted) (%) |  |  |  | <0.001 |
| No | 4566 (60.01) | 1877 (64.68) | 2689 (56.87) |  |
| Yes | 3192 (39.99) | 1080 (35.32) | 2112 (43.13) |  |
| MET, min/week, Mean (SD) | 3073.22 (5215.96) | 3108.67 (5281.52) | 3049.44 (5172.00) | 0.744 |
| Alcohol use, n (unweighted) (%) |  |  |  | 0.522 |
| No | 2735(30.74) | 1051 (30.17) | 1684 (31.12) |  |
| Yes | 5023 (69.26) | 1906 (69.83) | 3117 (68.88) |  |
| Hypertension, n (unweighted) (%) |  |  |  | <0.001 |
| No | 4968 (67.57) | 2299 (82.17) | 2669 (57.78) |  |
| Yes | 2790 (32.43) | 658 (17.83) | 2132 (42.22) |  |
| Diabetes, n (unweighted) (%) |  |  |  | <0.001 |
| No | 6796 (90.63) | 2836 (97.53) | 3960 (86.00) |  |
| Yes | 962( 9.37) | 121 ( 2.47) | 841 (14.00) |  |
| Nuts and seeds, g, Mean (SD) | 23.01 (48.51) | 26.46 (54.86) | 20.70 (43.58) | 0.001 |
| Whole fruits, g, Mean (SD) | 395.90 (448.76) | 434.00 (489.13) | 370.33 (417.63) | <0.001 |
| Whole grains, g, Mean (SD) | 90.65 (120.66) | 99.64 (126.85) | 84.61 (115.95) | <0.001 |
| Starchy vegetables, g, Mean (SD) | 119.45 (148.65) | 108.75 (140.37) | 126.63 (153.55) | <0.001 |
| Dairy, g, Mean (SD) | 479.02 (407.41) | 481.90 (399.16) | 477.08 (412.88) | 0.691 |
| Unsaturated oils, g, Mean (SD) | 46.53 (35.26) | 47.37 (35.98) | 45.96 (34.76) | 0.196 |
| Eggs, g, Mean (SD) | 50.79 (73.73) | 46.54 (70.69) | 53.65 (75.57) | 0.001 |
| Poultry, g, Mean (SD) | 82.91 (115.53) | 82.13 (112.40) | 83.44 (117.59) | 0.724 |
| Saturated oils, g, Mean (SD) | 79.12 (50.09) | 75.62 (48.68) | 81.47 (50.88) | <0.001 |
| Added sugar, g, Mean (SD) | 149.45 (126.44) | 149.59 (122.28) | 149.36 (129.17) | 0.954 |
| Legumes and soy food, g, Mean (SD) | 42.10 (100.27) | 41.89 (97.21) | 42.24 (102.28) | 0.907 |
| Nonstarchy vegetables, g, Mean (SD) | 328.32 (312.26) | 353.43 (382.79) | 311.47 (252.86) | <0.001 |
| Fish, g, Mean (SD) | 36.03 (89.30) | 36.50 (89.71) | 35.71 (89.04) | 0.733 |
| Red and processed meat, g, Mean (SD) | 160.36(155.11) | 141.85 (144.45) | 172.77 (160.70) | <0.001 |
| DGV/total ratio, Mean (SD) | 0.091 (0.173) | 0.106 (0.181) | 0.080 (0.167) | <0.001 |
| Rev/total ratio, Mean (SD) | 0.393 (0.262) | 0.388 (0.257) | 0.397 (0.265) | 0.191 |
| PHDI, Mean (SD) | 45.43 (13.56) | 47.32 (14.27) | 44.15 (12.90) | <0.001 |
